# LncRNA TP53TG1 plays an anti-oncogenic role in cervical cancer by synthetically regulating transcriptome profile in HeLa cells

**DOI:** 10.3389/fgene.2022.981030

**Published:** 2022-10-04

**Authors:** Yi Cheng, Nan Huang, Qingqing Yin, Chao Cheng, Dong Chen, Chen Gong, Huihua Xiong, Jing Zhao, Jianhua Wang, Xiaoyu Li, Jing Zhang, Shuangshuang Mao, Kai Qin

**Affiliations:** ^1^ Department of Oncology, Tongji Hospital, Tongji Medical College, Huazhong University of Science and Technology, Wuhan, Hubei, China; ^2^ Department of Allergy, Tongji Hospital, Tongji Medical College, Huazhong University of Science and Technology, Wuhan, Hubei, China; ^3^ Center for Genome Analysis, Wuhan Ruixing Biotechnology Co., Ltd., Wuhan, Hubei, China

**Keywords:** TP53TG1, apoptosis, cervical cancer, RNA-binding proteins, alternative splicing

## Abstract

Long non-coding RNAs (lncRNAs) have been extensively studied as important regulators of tumor development in various cancers. Tumor protein 53 target gene 1 (TP53TG1) is a newly identified lncRNA in recent years, and several studies have shown that TP53TG1 may play oncogenic or anti-oncogenic roles in different cancers. Nevertheless, the role of TP53TG1 in the development of cervical cancer is unclear. In our study, pan-cancer analysis showed that high expression of TP53TG1 was significantly associated with a better prognosis. We then constructed a TP53TG1 overexpression model in HeLa cell line to explore its functions and molecular targets. We found that TP53TG1 overexpression significantly inhibited cell proliferation and induced apoptosis, demonstrating that TP53TG1 may be a novel anti-oncogenic factor in cervical cancer. Furthermore, overexpression of TP53TG1 could activate type I interferon signaling pathways and inhibit the expression of genes involved in DNA damage responses. Meanwhile, TP53TG1 could affect alternative splicing of genes involved in cell proliferation or apoptosis by regulating the expression of many RNA-binding protein genes. Competing endogenous RNA (ceRNA) network analysis demonstrated that TP53TG1 could act as the sponge of several miRNAs to regulate the expression level of target genes. In conclusion, our study highlights the essential role of lncRNA TP53TG1 in the development of cervical cancer and suggests the potential regulatory mechanisms.

## 1 Introduction

Cervical cancer is the fourth most common malignancy diagnosed in women worldwide (Tsikouras et al., 2016). Nearly all cases of cervical cancer result from the infection of human papillomavirus (HPV) (Vu et al., 2018). In recent years, developed countries have adopted vaccination against HPV and conducted cervical screening with primary HPV testing followed by treatment of precancerous lesions, which is very effective in preventing and controlling the development of cervical cancer. However, cervical cancer incidence and mortality rates remain particularly high in developing countries, mainly in terms of chemotherapy resistance and metastasis (Tsikouras et al., 2016; Kori et al., 2019). It was already known that two main tumor suppressor proteins, p53 and retinoblastoma protein (pRb), are known to be inactivated by the HPV proteins, which disrupts both the DNA repair mechanisms and apoptosis, leading to rapid cell proliferation. Cell proliferation genes and Multiple genes involved in DNA repair become highly expressed in cervical cancer (Balasubramaniam et al., 2019). However, the molecular mechanisms of cervical cancer tumorigenesis and metastasis are far more complex. There is an urgent need to discover new accurate biomarkers and therapeutic targets.

Long non-coding RNAs (lncRNAs) have been reported to be important regulators of gene expression and innovative molecular biomarkers in cervical cancer (Silva et al., 2015). Previous studies reported that lncRNAs could act as competing endogenous RNAs (ceRNAs) or molecular sponges to interact with miRNAs and participate in the physiological and pathological process. For example, lncRNA TPT1-AS1 promotes cell growth and metastasis in cervical cancer *via* acting as a sponge for miR-324-5p which mediates the regulation of SP1 expression (Ji et al., 2019). SNHG7 regulated cervical cancer progression by sponging miR-485-5p, thereby up-regulating JUND expression (Zhao et al., 2020a). Furthermore, lncRNAs could interact with DNA, RNA, protein molecules and/or their combinations, acting as an essential regulator in chromatin organization, and transcriptional or post-transcriptional regulation (Yang et al., 2014). For example, lncAB bound KH-type splicing regulatory protein (KHSRP) and also decreased the expression of KHSRP in papillary thyroid carcinoma, thus increasing CDKN1a (p21) expression and decreasing CDK2 expression to repress cell proliferation (Gou et al., 2018). In addition, lncRNAs may function in alternative splicing by modulating splicing factors (Tripathi et al., 2010). The lncRNA 91H has been verified to regulate hnRNPK-mediated alternative splicing (AS), affecting cancer processes such as metastasis in CRC (Gao et al., 2018). To date, few studies in cervical cancer investigate the role of lncRNAs in post-transcriptional regulation in cervical cancer.

The lncRNA TP53TG1 (TP53 target gene 1) is a newly discovered p53-responsive lncRNA induced by DNA damage. It which exerts tumor-suppressive activities by inhibiting cell cycle progression, inducing apoptosis, or suppressing oncogene-induced transformation. Diaz-Lagares et al. (2016) found that the cancer growth suppressor features of TP53TG1 are linked to its ability to bind to RNA-binding protein YBX1 to prevent its nuclear localization and thus the YBX1-mediated activation of oncogenes (Diaz-Lagares et al., 2016). Several studies have suggested that TP53TG1 may act as an oncogene in different tumors. For instance, TP53TG1 promotes the growth and progression of pancreatic ductal adenocarcinoma by acting as a sponge to competitively bind to miR-96 and regulate KRAS expression (Aricò et al., 2019). TP53TG1 under glucose deprivation may accelerate cell proliferation and migration by influencing the expression of glucose metabolism-related genes in glioma (Gao et al., 2021). In spite of its bidirectional functions both in pro-tumor and anti-tumor effect, the role of TP53TG1 in cervical cancer remains unclear is not very clear. We also looked into whether TP53TG1 could be involved in transcriptional regulation and post-transcriptional control during the progression of cervical cancer.

To address the above-mentioned issues, we constructed a TP53TG1 overexpression model in HeLa cell line, coupled with RNA-seq sequences, to comprehensively reveal the biological functions of TP53TG1 in HeLa cell and analyze the alterations of transcriptional profiles upon TP53TG1 overexpression. In particular, we identified TP53TG1-regulated alternative splicing events (TP53TG1-RAS) and constructed a co-disturbed network between TP53TG1-RAS and RNA-binding protein genes, the expression of which is regulated by TP53TG1. An integrated “lncRNA/miRNA/mRNA” competing endogenous RNA (ceRNA) network was constructed to reveal potential regulatory relationships mediated by TP53TG1. Moreover we used LncSEA to analysis the biological function of TP53TG1 such as: epithelial mesenchymal transformation, immune, Autophagy/apoptosis, cell growth, coding ability, which may reveal that its biological function is very broad. Our study highlights the essential role of lncRNA TP53TG1 in the development of cervical cancer and discusses new regulatory mechanisms.

## 2 Materials and methods

### 2.1 Pan-cancer analysis of the cancer genome atlas (TCGA) data

The data of 24 cancer types from TCGA project, including gene expression profile and clinical information, were downloaded from the UCSC XENA database (https://xenabrowser.net/datapages/) to determine the expression levels of TP53TG1. Prognosis analysis of TCGA data was performed with GEPIA2 (Tang et al., 2017).

### 2.2 Single-cell RNA sequencing data preprocessing and analysis

Unique molecular identifier (UMI) count matrix of single-cell RNA-seq data from one cervical cancer tissue sample and one normal adjacent tissue sample was downloaded from GSE168652 (https://www.ncbi.nlm.nih.gov/geo/query/acc.cgi?acc=GSE168652). The 10x UMI count matrix was converted into a Seurat object using the R package Seurat (Butler et al., 2018) (version 4.0.4). Cells with UMI numbers <500 or with detected genes <200 or with over 15% mitochondrial-derived UMI counts were considered low-quality, and thus were removed. Genes detected in less than three cells were removed for downstream analysis.

After quality control, the UMI count matrix was log-normalized. Then the top 2,000 variable genes were used to create potential anchors with Find Integration Anchors function of Seurat. Subsequently, Integrate Data function was used to integrate data. To reduce the dimensionality of the scRNA-Seq dataset, principal component analysis (PCA) was performed on an integrated data matrix. With Elbowplot function of Seurat, the top 50 PCs were used to perform the downstream analysis. The main cell clusters were identified by the Find Clusters function offered by Seurat, with resolution set as default (res = 0.6). Finally, cells were clustered into 14 major cell types. Then they were visualized using tSNE or UMAP plots. To identify the cell type for each cluster, gene markers for each cell cluster were detected using the “Find Markers” function in Seurat package (v4.0.4). Then cell types were annotated using the cell markers provided in Gene Expression Omnibus (GEO) dataset (Ho et al., 2019).

### 2.3 Cloning and plasmid construction

pcDNA3.1-TP53TG1 (TP53TG1-overexpressing plasmid) was purchased from Youbio Biotech (Changsha, PRC). Full-length sequence of the plasmid:

ccc​tgt​ctc​cag​tgg​gcg​tct​tgg​gcc​ccg​gct​cta​ttc​tgg​gct​gcg​ggc​ctg​gga​agggct​cgc​cgg​gtg​cca​aat​gag​ctg​tcc​taa​ctc​tgc​ggg​gct​gca​gct​tcc​tgc​atg​atg​ctg​gggagc​ttg​gcg​cct​gac​cca​gga​tct​aga​agg​cac​tct​ggg​cag​gcc​gcg​ctc​cgc​cca​cga​agg​tac​cca​acc​ctc​tgg​gat​aga​tgc​agg​aag​cga​tgg​tta​aga​ccc​att​ttc​acc​caa​ctt​ctc​gcc​gca​ggt​ctg​gct​tac​cac​acg​ctc​ctc​ccc​att​ccc​agt​gag​ccg​ctt​ttt​gca​gca​cca​ggc​gaa​cac​tta​cac​cag​tgc​ttt​gta​aag​gaa​tct​tat​tgt​cca​ccc​cgt​gtc​ttg​gca​aaa​gaa​cag​tga​tca​cac​aga​ttc​cta​ctt​ggg​ctc​ttt​cct​tta​atc​ttc​gga​ggc​tga​gtt​tgc​cca​act​cag​gtttaa​cca​cca​agg​act​ctg​aga​gct​ggc​agg​tct​gag​taa​ccc​tgg​taa​caa​ttc​tct​tca​cct​tat​caa​aac​ctg​agc​taa​aac​caa​tgc​atc​agc​tga​tga​tga​cag​cag​aga​gtg​gca​ggg​ctg​agg​acc​caa​agt​cat​ttc​cca​ggc​tgg​cgg​aga​ata​aac​tgc​cag​gga​gaa​gaa​tga​gaa​gac​agg​agacaaactgtt​tgg​aaa​gct​aaa​tct​tcc​ctc​tta​atg​aat​aaa​ggt​ttt​tgc​ctt​gtc​tta​aaa​aaa​aa.

### 2.4 Assessment of TP53TG1 expression

GAPDH (glyceraldehyde-3-phosphate dehydrogenase) was used as a control gene for assessing the effects of TP53TG1 overexpression. cDNA was synthesized according to standard procedures and RT-qPCR was performed on the Bio-Rad S1000 with Hieff qPCR SYBR^®^ Green Master Mix (Low Rox Plus; YEASEN, China). The information of primers is presented in Additional File 1. The concentration of each transcript was then normalized to GAPDH mRNA level using 2^−ΔΔCT^ method (Livak and Schmittgen 2001).

### 2.5 Cell proliferation and apoptosis assay

The cell proliferation assay was conducted using a Cell Counting kit-8 (CCK-8; Dojindo Molecular Technologies, Inc., Shanghai, China). Briefly, TP53TG1-overexpressing Hela cells and control Hela cells were seeded with 6,000 cells/well in 96-well culture plates. Cells treated with an equal volume of phosphate-buffered saline (PBS) served as controls and vials without cells were used as blank controls. After incubated for 24, 48, and 72 h, 20 μl CCK-8 solution was added to the culture medium and incubated for an additional 3 h. Subsequently, the optical density (OD) of the cells was measured with a PerkinElmer/envision at an absorbance of 450 nm. The cell proliferation rate was calculated using the formula: proliferation rate = (experimental OD value − blank OD value)/(control OD value − blank OD value) × 100%.

#### 2.5.1 Apoptosis assay

For the flow cytometric analysis of cell apoptosis, the transfected cells were incubated at 37°C for 48 h and the living cells were then harvested and washed twice with ice-cold PBS. Viable cells were double-stained with 7-amino actinomycin D and FITC-conjugated Annexin V (Beijing 4A Biotech Co., Ltd.). The percentage of apoptotic cells was calculated as the sum of the right lower and upper quadrants. The number of stained cells was quantified using a flow cytometer (CytoFLEX; Beckman Coulter, Inc.). Cell cycle distribution was quantified using multi-cycle software (FlowJo 10.5.3; FlowJo, LLC).

### 2.6 RNA extraction and high-throughput sequencing

Total RNA was extracted using TRIzol reagent and was purified twice in phenol-chloroform. To remove DNA, the purified RNA was then treated with RNase-free RQ1 DNase (Promega Corp.) and its quality and quantity were determined by measuring the absorbance at 260 nm/280 nm (A260/A280) using a Smartspec Plus (Bio-Rad Laboratories, Inc.). The integrity of RNA was then verified by 1.5% agarose gel electrophoresis.

A total of 10 μg total RNA from each sample was used to prepare a directional RNA-seq library. First, the polyadenylated mRNAs were concentrated with oligo (dT)-conjugated magnetic beads (Invitrogen; Thermo Fisher Scientific, Inc.). The concentrated mRNAs were then iron-fragmented at 95°C, end-repaired and ligated to a 5′ adaptor. Reverse transcription (RT) was performed with RT primer harboring a 3′ adaptor sequence and randomized hexamer. The purified cDNAs were amplified and stored at −80°C for subsequent sequencing. Following the manufacturer’s instructions, the libraries were prepared for high-throughput sequencing. The Illumina Novaseq 6000 (Illumina, Inc.) was used to collect data from 150-bp paired-end sequencing (Illumina, Inc.).

### 2.7 RNA-seq raw data cleaning and alignment

Raw sequencing reads containing more than 2-N bases were first discarded. Subsequently, adaptors and low-quality bases were trimmed off the raw reads using a FASTX-Toolkit (v.0.0.13; http://hannonlab.cshl.edu/fastx toolkit/). Short reads of less than 16 nt were dropped. Then clean reads were subsequently aligned to the GRch38 genome by HISAT2 (Kim et al., 2015). Uniquely mapped reads were ultimately used to calculate read number and paired-end fragments per kilobase of exon per million fragments mapped (FPKM) for each gene.

### 2.8 Differentially expressed gene analysis

The gene expression levels were measured using FPKM. The DEGs were screened using the software DEseq2 (Love et al., 2014), which analyzes DEGs. Using the fold change (FC ≥ 2 or ≤0.5) and false discovery rate (FDR<0.05) as the cutoff, the results were analyzed to determine whether a gene was differentially expressed.

### 2.9 Alternative splicing analysis

The alternative splicing events (TP53TG1-AS) and regulated alternative splicing events (TP53TG1-RAS) between TP53TG1 overexpression (TP53TG1-OE) samples and control samples were defined and quantified using the SUVA pipeline described previously (Cheng et al., 2021a). The frequency and proportion of reads of SUVA AS events (pSAR) were calculated.

### 2.10 CeRNA network construction

TP53TG1-mediated ceRNA network. Miranda (score ≥ 150) and Rnahybrid (*p*-value ≤ 0.05) were used to predict the target relationship between miRNA and TP53TG1. Finally, the results of the two methods were intersected. miRNA-mRNA target pairs from miRDB (http://mirdb.org) and targetscan (http://www.targetscan.org/vert_80/) databases were used to predict the target relationship between miRNA and TP53TG1-regulated DEGs. The regulatory network was established using Cytoscape (https://cytoscape.org/).

### 2.11 Functional enrichment analysis

Using the KOBAS 2.0 server (Xie et al., 2011), GO analyses and enriched Kyoto Encyclopedia of Genes and Genomes (KEGG) pathways were adopted to predict the functions of genes and calculate the distribution frequency in each functional category. The enrichment of each pathway (corrected *p* < 0.05) was defined using hypergeometric tests and the Benjamini-Hochberg FDR controlling procedure.

### 2.12 Statistical analysis

One-way analysis of variance and GEPIA2 were used for comparison of expression levels of TP53TG1 among 24 TCGA cancer types, with disease state (Tumor or Normal) as the variable for calculating differential expression. Student’s *t*-test was used for all other comparisons between the TP53TG1-OE and control groups. For each assay, the results are presented as the mean ± standard error of the mean values of three experiments. The data were analyzed using R software (v3.5.3, https://www.r-project.org/). *p* < 0.01 or FDR<0.05 was set as the threshold to indicate a statistically significant difference.

## 3 Results

### 3.1 Pan-cancer analysis shows that high TP53TG1 expression is associated with a good prognosis

**FIGURE 1:**
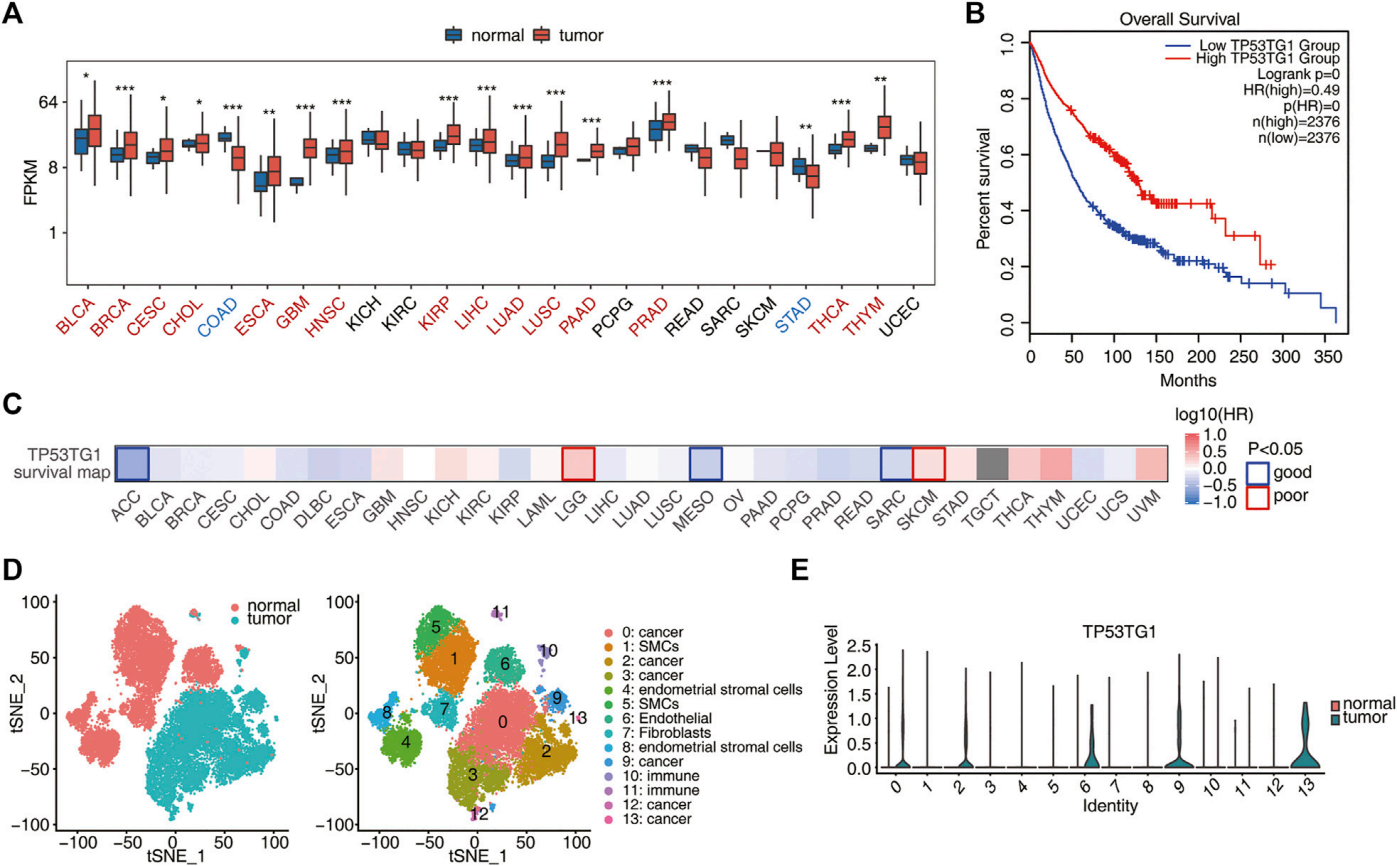
Pan-cancer analysis shows that high expression of TP53TG1 is associated with a good prognosis. **(A)** Relative expression (FPKM) of TP53TG1 in tumor samples (red) compared with in normal samples (blue) in 24 cancer types from TCGA. *p < 0.05; **p < 0.01; ***p < 0.001. **(B)** Association of TP53TG1 expression with the survival rates is elucidated for comparison between upper quartile and lower quartile patients from TCGA pan-cancer dataset. **(C)** Association of TP53TG1 expression with the survival rates in various types of cancer. **(D)** Single-cell RNA-seq data from one cervical cancer tissue sample and one normal adjacent tissue sample were downloaded from GSE168652, respectively. Cell types with cell cluster id for tSNE plot are summarized in the right panel. **(E)** Violin plot showing the expression alternation of TP53TG1 between tumor and normal samples in different cell clusters.

Inspired by previous discoveries that TP53TG1 may play oncogenic or anti-oncogenic roles in different cancers (Zhang et al., 2019b; Islam et al., 2021), TP53TG1 expression was compared between tumor and normal tissues in 24 cancer types from TCGA. TP53TG1 expression in tumors was significantly up-regulated (*p-value* < 0.05) in 15 of the 24 cancer types and was significantly down-regulated only in two cancer types, including colorectal and gastric cancer (Figure 1A). This is consistent with that p53 transcriptionally activates TP53TG1 when the DNA-damaging agent is used except that tumor-specific promoter CpG island hypermethylation-associated silencing of the lncRNA TP53TG1 occurs in colorectal and gastric cancer cells (Diaz-Lagares et al., 2016). Furthermore, GEPIA2 was used to comprehensively analyze the association of TP53TG1 expression with survival rates in 2,376 patients. Pan-cancer analysis shows that higher TP53TG1 expression was positively correlated with a higher overall survival rate (Figure 1B), which confirmed that TP53TG1 exhibits tumor suppressor-like feature in cancer patients (Diaz-Lagares et al., 2016). Furthermore, the association of TP53TG1 expression with survival rates in various types of cancer was also depicted using the survival map (Figure 1C). Surprisingly, we found that TP53TG1 showed positive or negative correlations with prognosis in different cancers, and that this correlation was significant in only five of them. Taken together, these results suggest that TP53TG1 may have a dual role in cancer development, with a predominant oncogenic function. Its function is associated with regulatory networks in cancerous tissue.

For cervical cancer (CESC), the expression of TP53TG1 in tumor tissue was higher than that in adjacent normal tissue (Figure 1A; Supplementary Figure S1A). Prognostic analysis showed that high expression of TP53TG1 was associated with a good prognosis (Figure 1B; Supplementary Figure S1B). Since bulk RNA-seq obtains the average gene expression of various cells, to know whether TP53TG1 is highly expressed in malignant cells or in other cell types, we downloaded and analyzed the single cell data of cervical cancer tissue and adjacent tissue, respectively. (GSE168652) (Li et al., 2021) (Figure 1D). We found that TP53TG1 was highly expressed in cell types C0 (cancer cells), C2 (cancer cells), C6 (endothelial cells), C9 (cancer cells) and C13 (cancer cells) (Figure 1E). These results reveal that TP53TG1 is activated in many malignant tumors and has a potential inhibitory effect on cancer development, resulting in a better prognosis.

### 3.2 TP53TG1 overexpression significantly inhibits proliferation and promotes apoptosis of HeLa cells

**FIGURE 2:**
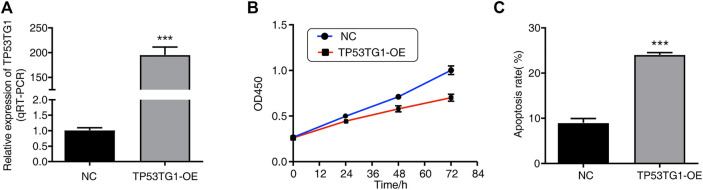
TP53TG1 over-expression significantly inhibits proliferation and promotes apoptosis of HeLa cells. **(A)** Relative expression of TP53TG1 was validated by RT-qPCR in HeLa cells after it was overexpressed. **(B)** The cell proliferation index was calculated according to the OD450 value. **(C)** Apoptosis of TP53TG1-overexpressing HeLa cells and controls was measured using flow cytometry.

To further investigate the function of TP53TG1 in cervical cancer, a TP53TG1-overexpression (TP53TG1-OE) cell model was constructed by transfecting a TP53TG1-overexpression plasmid into HeLa cells, with empty vector as negative control (NC). The overexpression of TP53TG1 was verified by RT-qPCR. The result showed that TP53TG1 gene expression significantly increased in TP53TG1-OE group (*p* < 0.001; Figure 2A). To characterize the role of TP53TG1 in regulating cell apoptosis and proliferation in HeLa cells, flow cytometric analyses and CCK8 assay were performed, respectively. Cell proliferation significantly decreased in the TP53TG1-OE group after 24 h (*p* < 0.01; Figure 2B; Supplementary Figure S2A). Meanwhile, TP53TG1-OE significantly promoted cell apoptosis (*p* < 0.001; Figure 2C). Together, these results indicated that TP53TG1 plays a tumor suppressor role in the progression of Hela cells.

### 3.3 TP53TG1 regulates the expression of genes involved in type I interferon signaling pathways and DNA damage responses

**FIGURE 3:**
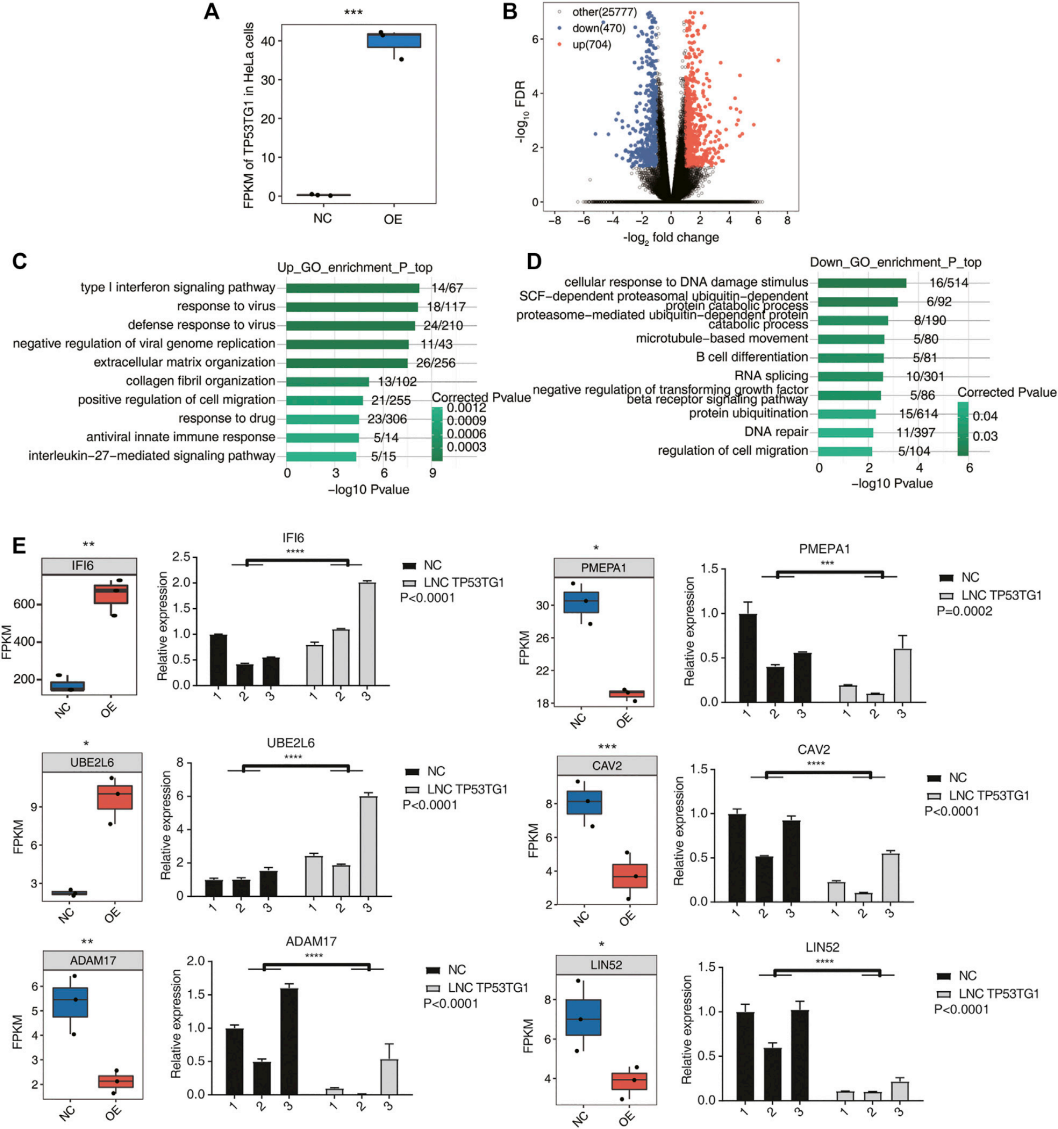
TP53TG1 regulates the expression of genes involved in type I interferon signaling pathways and DNA damage responses. **(A)** Boxplot showing the expression pattern of TP53TG1 in TP53TG1-overexpressing (TP53TG1-OE) HeLa cells and controls. **(B)** Volcano plot presenting the differences between TP53TG1-OE and NC groups. The results showed the number of differences between TP53TG1-OE group and control group (FC ≥ 2 or ≤0.5 and FDR ≤ 0.05. **(C,D)** The bar plot exhibiting the most enriched GO biological process results of the upregulated **(C)** and downregulated **(D)** DEGs. **(E)** Expression profile of DEGs regulated by TP53TG1 in RNA-seq along with in reverse transcription-qPCR validation in HeLa cells. Black bars represent the control group and grey bars represent TP53TG1 overexpression. ***p < 0.001.

LncRNAs participate in the regulation of gene expression and play vital roles in various biological and pathological processes (Liao et al., 2016). To investigate TP53TG1-mediated transcriptional or post-transcriptional regulation in HeLa cells, cDNA libraries for TP53TG1-OE cells and control cells were constructed for RNA-seq using the Illumina HiSeq Novaseq 6000 platform. Three biological replicates for each group were used and a total of 113–135 Million (M) 150-nucleotide paired-end raw reads per sample were obtained. After removing adaptors and low-quality reads, 103.5–125.4 M clean reads were aligned to the human GRCH38 genome using HISAT2, of which 86.27%–90.24% were uniquely aligned for further analysis (Supplementary Table S1). The level of gene expression was calculated in the units of FPKM (Supplementary Table S2). A total of 26,951 expressed genes were assessed by RNA-seq. Effective overexpression of TP53TG1 was further confirmed by the use of RNA-seq analysis (Figure 3A). The differentially expressed genes (DEGs) between the TP53TG1-OE and control cells were determined using an absolute FC ≥ 2 and a 5% FDR as criteria coupled with the DESeq2 package (Love et al., 2014). A total of 704 up-regulated and 470 downregulated DEGs were identified (Supplementary Table S3). The DEGs associated with TP53TG1-OE are displayed in a volcano plot (Figure 3B).

To further explore the potential biological roles of these DEGs, GO (Supplementary Table S4) and KEGG (Supplementary Table S5) enrichment analyses were performed. The top 10 GO terms in the category biological process were shown in Figure 3C and Figure 3D. And the top 10 KEGG terms in the category biological process were shown in Supplementary Figures S3A,B. The upregulated genes in the TP53TG1-OE cells are mainly enriched in type I interferon signaling pathways, response to virus, and positive regulation of cell migration terms (Figure 3C). Previously, type I interferon was reported to inhibit the growth of tumor stem cells and promote the apoptosis of cancer cells (Bekisz et al., 2010; Aricò et al., 2019). The downregulated genes were mostly associated with DNA damage responses, such as cellular response to DNA damage stimulus and DNA repair. Genes involved in RNA splicing and regulation of cell migration were also significantly enriched in downregulated genes (Figure 3D). DNA damage is closely related to apoptosis in cancer (Zhang et al., 2007; Figueroa-González and Pérez-Plasencia, 2017). These results indicated that TP53TG1 could active the type I interferon signaling pathways and suppress DNA damage responses in HeLa cells, which ultimately promotes the apoptosis of cancer cells.

To confirm the regulatory function of TP53TG1 in gene expression in the HeLa cell line, several DEGs were involved in type I interferon signaling pathways, cellular response to DNA damage stimulus and DNA repair, which were validated by RT-qPCR (Figure 3E). And these genes might be related to prognosis (Supplementary Figure S3C).

### 3.4 TP53TG1 regulates the alternative splicing of genes associated with proliferation and apoptosis processes and the expression of a large number of RBP genes

**FIGURE 4:**
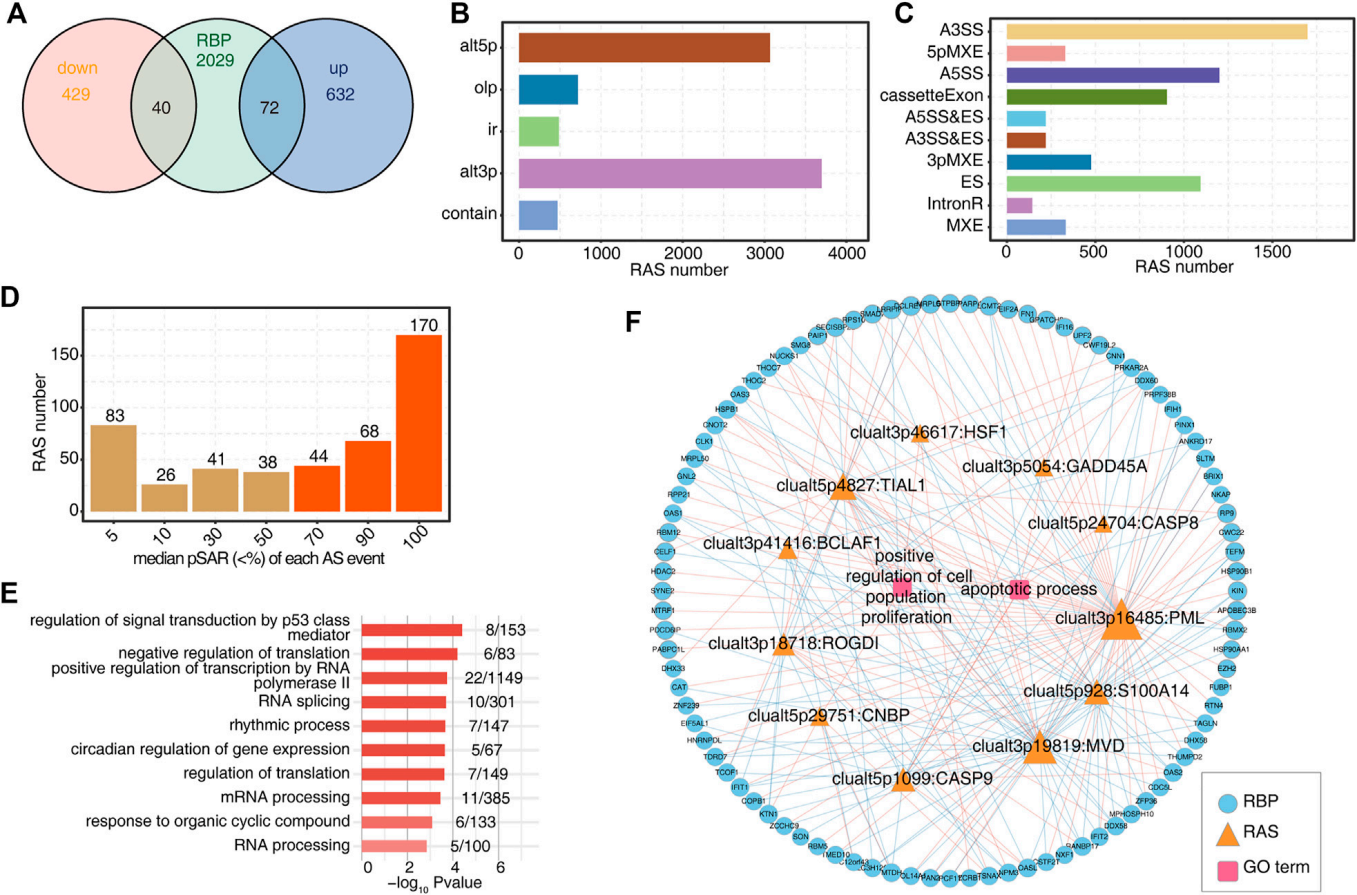
TP53TG1 regulates the alternative splicing of genes associated with proliferation and apoptosis and the expression of a large number of RBP genes. **(A)** Venn diagram showing the number of DEGs regulated by TP53TG1 and RNA-binding proteins. **(B)** Bar plot shows the number of RAS events detected by SUVA between TP53TG1-OE and control samples. **(C)** Splice junction constituting TP53TG-regulated RAS events was annotated as classical AS event types. And the number of each classical AS event type was shown in the bar plot. **(D)** Bar plot showing TP53TG-regulated RAS events with different pSAR. RAS events whose pSAR (Reads proportion of SUVA AS event) ≥50% were labeled in orange and were used for further analysis. **(E)** GO enrichment analysis of the biological processes of genes with TP53TG-regulated RAS events (RASG) whose pSASR ≥ 50%. **(F)** The co-disturbed network among expression of DE RBPs and splicing ratios of RAS events (pSAR ≥ 50%) which located on proliferation and apoptosis-related genes was constructed. |Pearson’s correlation| ≥0.95 and *p*-value ≤ 0.01 were retained for RBP and RAS correlation. Circles represent RBP genes. Triangles indicate RAS. Squares in center indicate GO terms. Node size indicates the degree of connection with other nodes.

LncRNAs were shown to interact with transcriptional factors and thus affect transcription. They are also able to regulate the alternative splicing (AS) by interacting with splicing factors (SFs) or other RNA-binding proteins (RBPs) (Zhang et al., 2019a). Inspired by these previous discoveries, we compared the DEGs with reported RBP gene lists (Castello et al., 2012; Gerstberger et al., 2014; Castello et al., 2016; Hentze et al., 2018). We found that 72 RBP genes were upregulated and 40 RBP genes were downregulated in TP53TG1-OE cells (Figure 4A). Herein we propose that TP53TG1 could regulate alternative splicing by modulating the expression of RBPs. We used the recently published AS analysis software SUVA to identify AS events significantly altered between TP53TG1-OE and NC group (TP53TG1-RAS).

As shown in Figure 4B, by applying a stringent cut-off of *p* ≤ 0.05 and an AS ratio ≥0.2, 470 high-confidence RASEs were identified (Supplementary Table S8) (Cheng et al., 2021a). There are five RAS types: 249 alt3p, 156 alt5p, 13 contain, 14IR and 38 Olp. The splicing events were corresponding to classical splicing events, in which A3SS events accounted for a large proportion (Figure 4C), which may be one of the characteristics of TP53TG1-RAS. As shown in Supplementary Figure S4B, Alt3p and alt5P events, A3SS and A5SS events dominate the alternative splicing events. Further, about 60% of TP53TG1-RAS events were complex splicing events (Supplementary Figures S4C,D), indicating the complexity of AS regulation by TP53TG1. A splicing event involves two transcripts, and these two transcripts may only account for a very small part of the expression of the whole gene. We hope to find a more dominant transcript undergo AS which was quantified as “pSAR” value by SUVA. Here principal component analysis (PCA) was conducted by using the splicing ratio of different variable splicing events, and it could be seen that the principal components of the two groups could be well separated. And the samples’ correlation is strong (Supplementary Figure S4E). The number of splicing events accounting for different pSAR was shown (Figure 4C). Finally, 470 main splicing events with pSAR ≥ 50% were selected for further analysis (Figure 4D). As shown in Supplementary Figures S4C,D, the TP53TG1-OE group and the NC group can be clearly distinguished by the splicing ratio of TP53TG1-RAS (pSAR ≥ 50%).

Overall, genes linking with TP53TG1-RAS were primarily enriched in the proliferation, apoptosis (Supplementary Table S7) and signal transduction by P53 mediator pathways (Figure 4E). As shown in Figure 4F, the expression of differentially expressed RBPs and the splicing ratio of TP53TG1-RAS events associated with proliferation and apoptosis were used for correlation analysis (Pearson’s correlation coefficient ≥0.8, *p* ≤ 0.01). These RBPs might potentially regulate TP53TG1-RAS (Supplementary Figure S4F). The size of nodes in the figure represents the number of gene/splicing events associated with them, including two intron retention (IR), one A5SS, two ES and 1 CE which were located on BCLAF (Supplementary Figure S4G).

### 3.5 The alternative splicing events are regulated by TP53TG1

**FIGURE 5:**
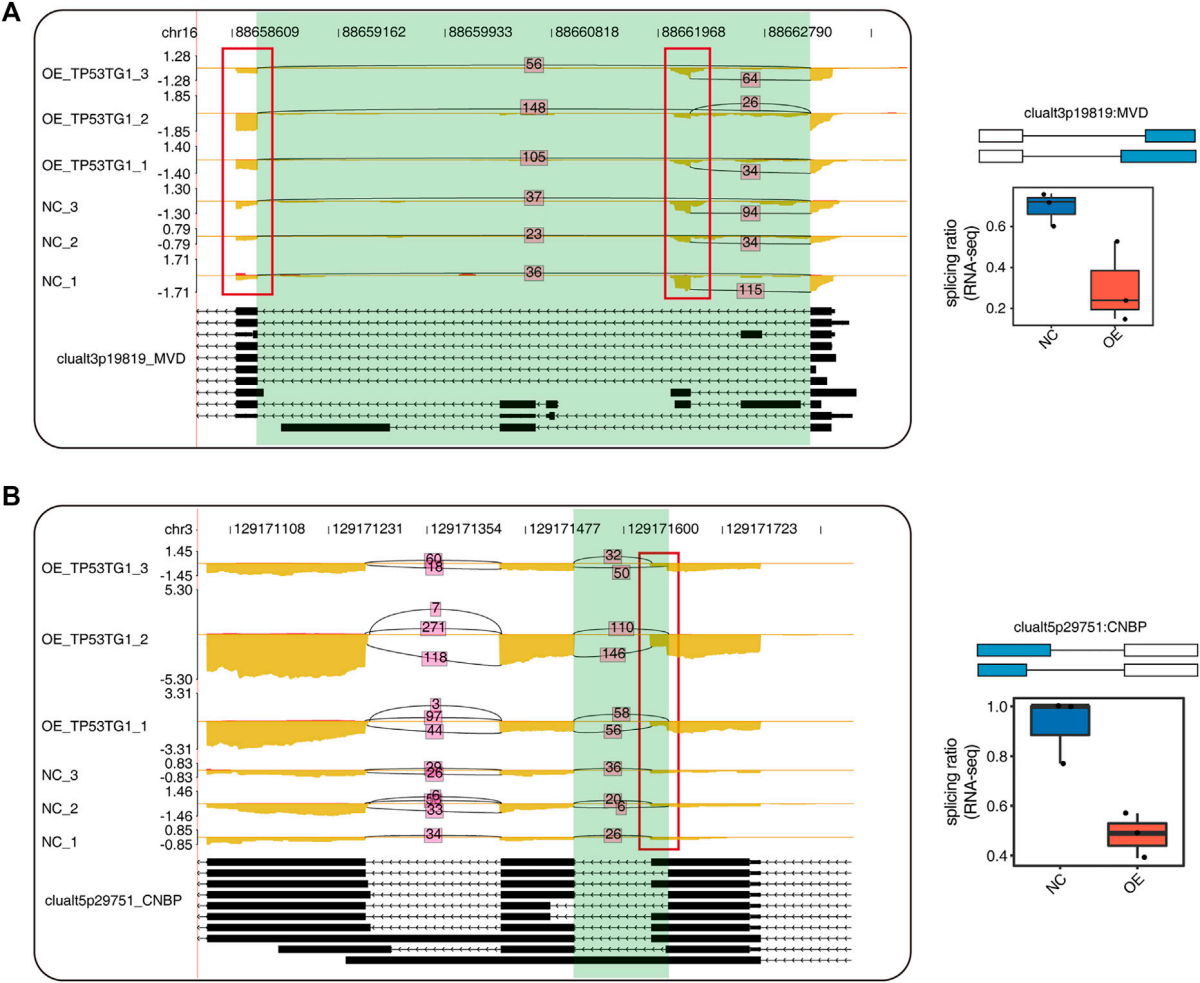
The Alternative splicing events which regulated by TP53TG1. **(A)** Visualization of junction reads distribution of clualt3p19819 RAS event located on MVD in samples from different groups. Splice junctions were labeled with SJ reads number. And altered splice site was marked out with red box. Splicing events model was shown in the right-up panel. Splicing ratio profile of RNA-seq along with reverse transcription-qPCR validation in HeLa cells were shown in the right-bottom panel. **(B)** Visualization of junction reads distribution of clualt5p29751 RAS event located on CNBP in samples from different groups. Splice junctions were labeled with SJ reads number. And altered splice site was marked out with red box. Splicing events model was shown in the right-up panel. Splicing ratio profile of RNA-seq in HeLa cells were shown in the right-bottom panel.

Here represents the number of gene/splicing events associated with them, including two intron retention (IR), one A5SS, two ES and 1 CE which were located on MVD, CNBP (Figures 5A,B).

### 3.6 The regulatory network of lncRNA-miRNA-DEG mediated by TP53TG1 in HeLa cells

**FIGURE 6:**
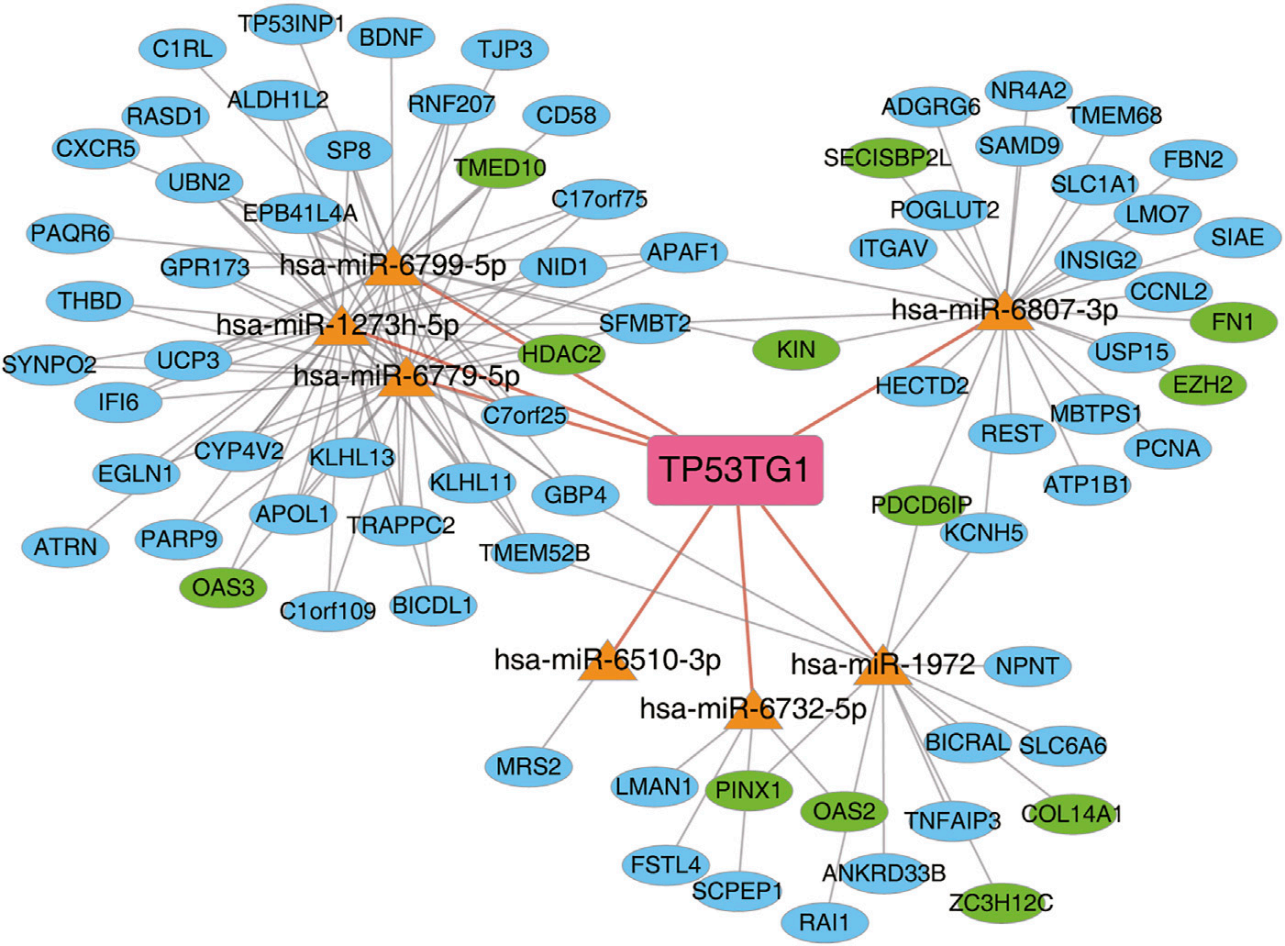
The regulatory network of lncRNA-miRNA-DEG mediated by TP53TG1 was deregulated in HeLa cells. TP53TG1-mediated ceRNA network. Miranda and Rnahybrid were used to predict the target relationship between miRNA and TP53TG1. miRNA-mRNA target pairs from miRDB (http://mirdb.org) and targetscan (http://www.targetscan.org) databases were used to predict the target relationship between miRNA and TP53TG1-regulated DEGs.

CeRNAs have emerged as an important mechanism for lncRNA and miRNA regulatory network (Su et al., 2021). To investigate TP53TG1-mediated ceRNA network, miranda (Enright et al., 2003) and rnahybrid (Rehmsmeier et al., 2004) were used to predict the target relationship between miRNA and TP53TG1, respectively. Then, seven overlapping miRNAs, containing has-miR-6799-5p, has-miR-1273h-5p, has-miR-6779-5p, has-miR-6807-3p, has-miR-6510-3p, has-miR-6732-5p, and has-miR-1972, were screened. The seven miRNAs were mapped into miRDB (http://mirdb.org) and targetscan (http://www.targetscan.org) databases to explore their target genes, accompanied with TP53TG1-regulated DEGs. Finally, integrating the TP53TG1/miRNA interactions with the miRNA/DEGs interactions, a ceRNA network was constructed (Figure 6). The result reveals that lncRNA-TP53TG1 could adsorb miRNAs as a sponge RNA. The predicted ceRNA network comprised 426 nodes (13 miRNAs and 413 mRNAs) and 743 interactions. In this network (Figure 6), several nodes and interactions involving the feature genes play essential roles in CESC, such as hsa-miR-6779-5p (target genes: OAS3, HDAC2 and IFI6), hsa-miR-1972 (target genes: PINX2, OAS2, ZC3H12C), and hsa-miR-6807-3p (target genes: PDCD6IP, KIN, and EZH2), and all these target genes were upregulated. MiR-6779-5p was also identified as a prognosis-related miRNA (Yang et al., 2022).

## 4 Discussion

In this study, we investigated the role of TP53TG1 in cervical cancer and explored whether TP53TG1 could function in transcriptional or post-transcriptional regulations. First, by analyzing Pan-cancer data from TCGA, a total of 17 out of 24 cancer types exhibited upregulation of TP53TG1 and only two cancer types exhibited downregulation. High expression of TP53TG1is associated with a good prognosis. Single-cell data and bulk RNA-seq analysis revealed that TP53TG1 is mainly activated in malignant cells and has a potential inhibitory effect on CESC cells, suggesting that TP53TG1 may play an important role in CESC development. On the basis of these findings, the HeLa cell line was used as a model to analyze the consequences of TP53TG1 overexpression. The overexpression of TP53TG1 promoted cell apoptosis and inhibited cell proliferation. Transcriptome sequencing demonstrated that the overexpression of TP53TG1 had a broad effect on gene expression. Functional clusters of DEGs highly enriched in cancer-associated terms were obtained. The upregulated DEGs were enriched in the type I interferon pathway and the downregulated DEGs were enriched in DDR pathway. Furthermore, overexpression of TP53TG1 significantly regulated the AS of genes involved in the mRNA splicing, apoptotic process and DNA repair pathways mediated by the expression alternation of dozens of TP53TG-regulated RBPs. Finally, a predicted ceRNA network was constructed.

TP53TG1, known as a p53-induced lncRNA promotes apoptosis of cancer cells. In colorectal and gastric cancer, TP53TG1 is downregulated due to DNA methylation (Diaz-Lagares et al., 2016). Consistently, we found that the expression of TP53TG1 is up-regulated in most cancers except in colorectal and gastric cancers, perhaps due to chemotherapy and DNA methylation. Pan-cancer analysis also shows that higher expression of TP53TG1 was associated with a better prognosis, indicating that TP53TG1 inhibits the progression of cancer. However, TP53TG1 may act as a tumor suppressor gene or an oncogene in different tumors (Zhang et al., 2019b; Lu et al., 2021). It functions as an oncogene in pancreatic ductal adenocarcinoma (PDAC), retinoblastoma, and nasopharyngeal carcinoma (Yuan et al., 2017; Zhang et al., 2019b; Shao et al., 2020; Tang et al., 2020; Cheng et al., 2021a; Gao et al., 2021; Lu et al., 2021; Wang et al., 2021). In some other cancers, such as non-small cell lung cancer, hepatocellular carcinoma, gastrointestinal cancer, and cutaneous melanoma, TP53TG1 serves as a cancer suppressor (Benfodda et al., 2018; Xiao et al., 2018; Chen et al., 2021; Masoumi et al., 2021). Through TCGA analysis, expression of TP53TG1 is positively or negatively correlated with overall survival time in different cancers (Zhang et al., 2019b). These findings suggest that TP53TG1 plays a dual regulatory function in cancer and that its function varies in different cancer types and environment. TP53TG1 has been reported to be cancer-promoting in cervical cancer (Liao et al., 2022), but prognosis analysis showed its cancer-inhibiting function. Moreover, we also performed single cell RNA-seq analysis of tumor and adjacent normal tissue and found for the first time that TP53TG1 is predominantly expressed in cancer cells.

Previous studies reported that the high expression of TP53TG1 inhibited cancer cell apoptosis in PDAC and breast cancer (Zhang et al., 2019b; Shao et al., 2020) and promoted cancer cell apoptosis in non-small cell lung cancer, hepatocellular carcinoma, gastrointestinal cancer, and cutaneous melanoma (Benfodda et al., 2018; Xiao et al., 2018; Chen et al., 2021; Masoumi et al., 2021). In this study, we constructed a TP53TG1-OE model in HeLa cell line to explore its role in cervical cancer. Conclusively, TP53TG1 significantly promotes apoptosis in Hela cells, inconsistent with existing reports that TP53TG1 inhibits apoptosis in cervical cancer (Liao et al., 2022). Whereas, the results we obtained were consistent with the results of the prognostic analysis. Previous studies have reported that TP53TG1 has a dual role in cancer (Lu et al., 2021). We hypothesized that TP53TG1 may have both tumor-suppressing and tumor-promoting effects in different cells under different conditions. In the future, we will explore the functions of TP53TG1 in more cell lines.

Over the last decade, type I interferons (IFNs) have been studied extensively for inducing apoptosis in tumor cells (Pokrovskaja et al., 2005). IFN treatment could induce the tumor suppressor gene p53 (Bekisz et al., 2010). Furthermore, IFNs can up-regulate caspase-4 and caspase-8, which can activate the initiator caspases-8 and 9, as well as the effector caspase-3, USP18 and STAT3 in increased sensitivity of cells to pro-apoptotic (Matsui et al., 2003, Schreiber and Piehler 2015; Thyrell et al., 2002). Currently, upregulated gene of TP53TG1-OE were mostly enriched in type I interferon signaling pathway, which may be the direct reason for its pro-apoptotic effect. The activation of IFNs may induce p53 and further induce the expression of TP53TG1 to form a positive cycle. Next, we compared the RNA-seq data in cancer cells with those in clinical samples. We found five up-regulated expression IFNI-associated genes such as IFI6, OAS1, OAS3, IFIT1 and OASL. These five upregulated genes are generally associated with tumorigenesis and progression, including impressed cell proliferation and induced apoptosis (Matsui et al., 2003; Mullan et al., 2005; Hovanessian, 2007; Niu et al., 2016; Boehmer et al., 2021). Interestingly, overexpression of TP53TG1 also upregulates the expression of genes which promote cell migration, indicating the beneficial effect of TP53TG1 on cancer cell metastasis, this finding being consistent with the previous report that TP53TG1 may act as a tumor suppressor gene or an oncogene in different tumors (Zhang et al., 2019b).

DNA damage response (DDR) is crucial for maintaining genome stability in cancer cells (Yoshida and Miki 2004). TP53TG1 is a lncRNA that is critical for the correct response of p53 to DNA damage (Diaz-Lagares et al., 2016). This prompted us to investigate how TP53TG1 regulates the DDR pathway in cancer cells, which could affect the sensitivity of chemotherapy drugs. Surprisingly, we found that genes involved in DDR pathways were significantly downregulated in/by the overexpression of TP53TG1. The overexpression of TP53TG1 could significantly reduce the DNA damage repair efficiency in cancer cells, so as to promote apoptosis of cancer cells. These gene, such as FOXP1, YY1, USP28, NUCKS1, and PCLAF were related to DDR pathway. PCLAF is highly expressed in neuroblastoma, which can accelerate neuroblastoma cell proliferation and cell cycle progression and restrain cell apoptosis (Liu et al., 2022). These genes are generally associated with tumorigenesis and progression, including induced cell proliferation and inhibited apoptosis (Wu et al., 2018; Zhao et al., 2019; Zhao et al., 2020b; Liu et al., 2020). Their downstream targets are regulated by TP53TG1 and may influence cancer progression in cells or clinical samples.

The mechanism of lncRNAs regulating AS involved interactions with RNA-binding proteins especially with splicing factors, which control AS through the interaction with pre-RNA sequences (Gordon et al., 2019). TP53TG1 binds to RNA-binding protein YBX1 to block its function and acts as an anti-cancer agent (Diaz-Lagares et al., 2016). In this study, we found that TP53TG1 regulates the expression of a large number of RBPs and we speculated that TP53TG1 may play a prominent role in post-transcriptional regulation, such as in alternative splicing, by regulating the expression of RBPs. We are particularly concerned that the alternative splicing of the p53 pathway could prevent and repair damaged DNA and create feedback loops that enhance or attenuate p53 activity and communicate with other signal transduction pathways. Some proliferation- and apoptosis-related genes are significantly regulated by TP53TG1. These genes could provide a new mechanism for TP53TG1 in post transcriptional regulation. Meanwhile, we also established a ceRNA network based on predicted interactions among TP53TG1, miRNAs, and DEGs. TP53TG1 is predicted to serve as a sponge of miRNAs in gene expression. In the future research, we aspire to discover how TP53TG1 regulates the expression of important genes and RBPs, thereby affecting the splicing.

## Data Availability

The datasets presented in this study can be found in online repositories. The name of the repository and accession number can be found below: NCBI; GSE206471.
