# Supplementary material for: LncRNA TP53TG1 plays an anti-oncogenic role in cervical cancer by synthetically regulating transcriptome profile in HeLa cells
Source: Front Genet. 2022 Oct 4;13:981030. doi: 10.3389/fgene.2022.981030 (PMC9576931; doi:10.3389/fgene.2022.981030)
Supplement: Supplementary file 2 [file Presentation1.pdf]

Supplementary Material

Supplementary Figures

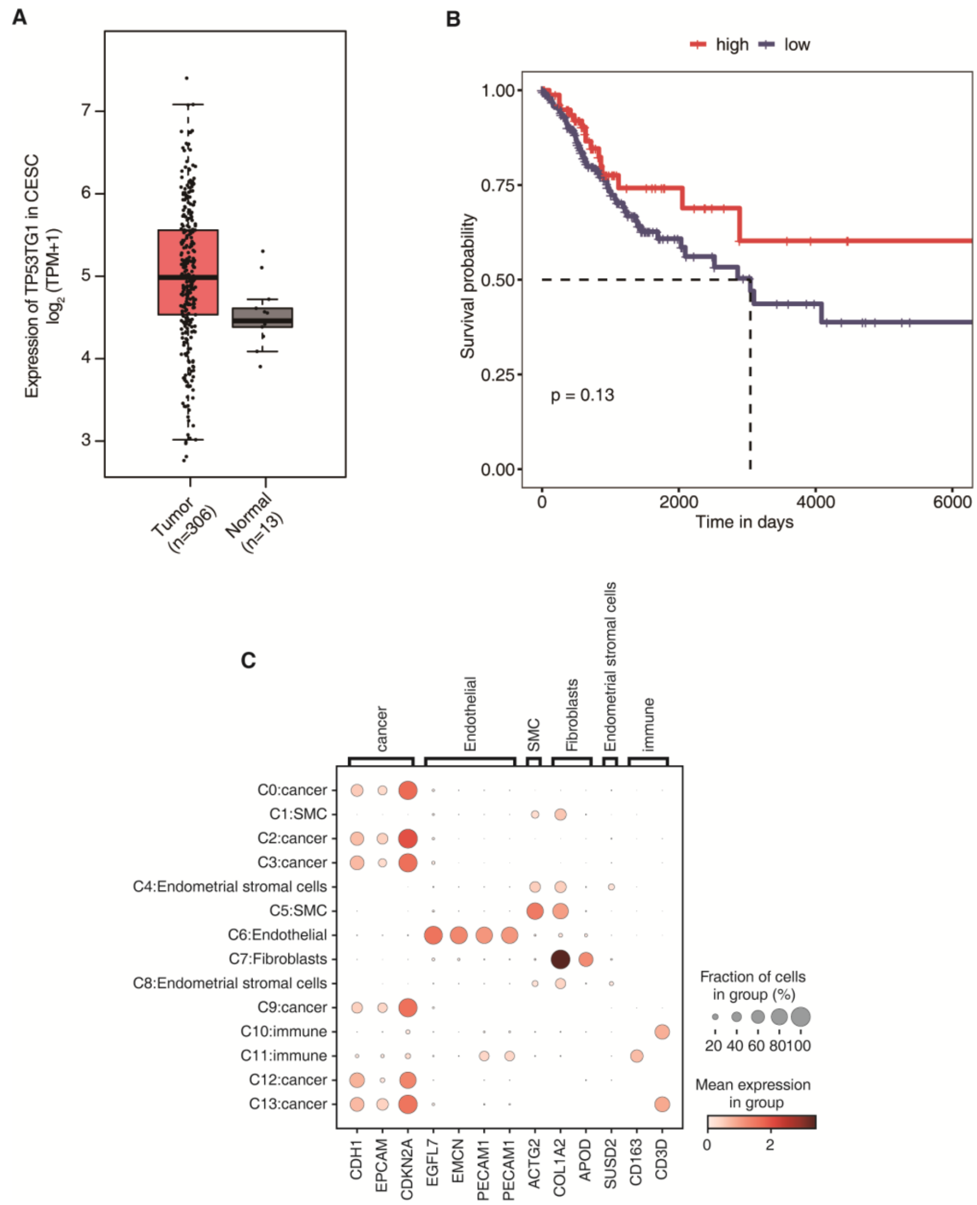

Supplementary Figure 1. Pan-cancer analysis shows that high TP53TG1 expression is associated with a good prognosis. **(A)** Box plot showing the relative expression of TP53TG1 in tumor samples (red) of CESC patients compared with in normal samples (grey) from the TCGA database. ***P<0.001. (B) Overall survival (OS) analysis of TP53TG1 in 293 analyzed CESC patients from TCGA with high versus low expression.(C) Dot plot showing the expression of representative genes in each cell type.

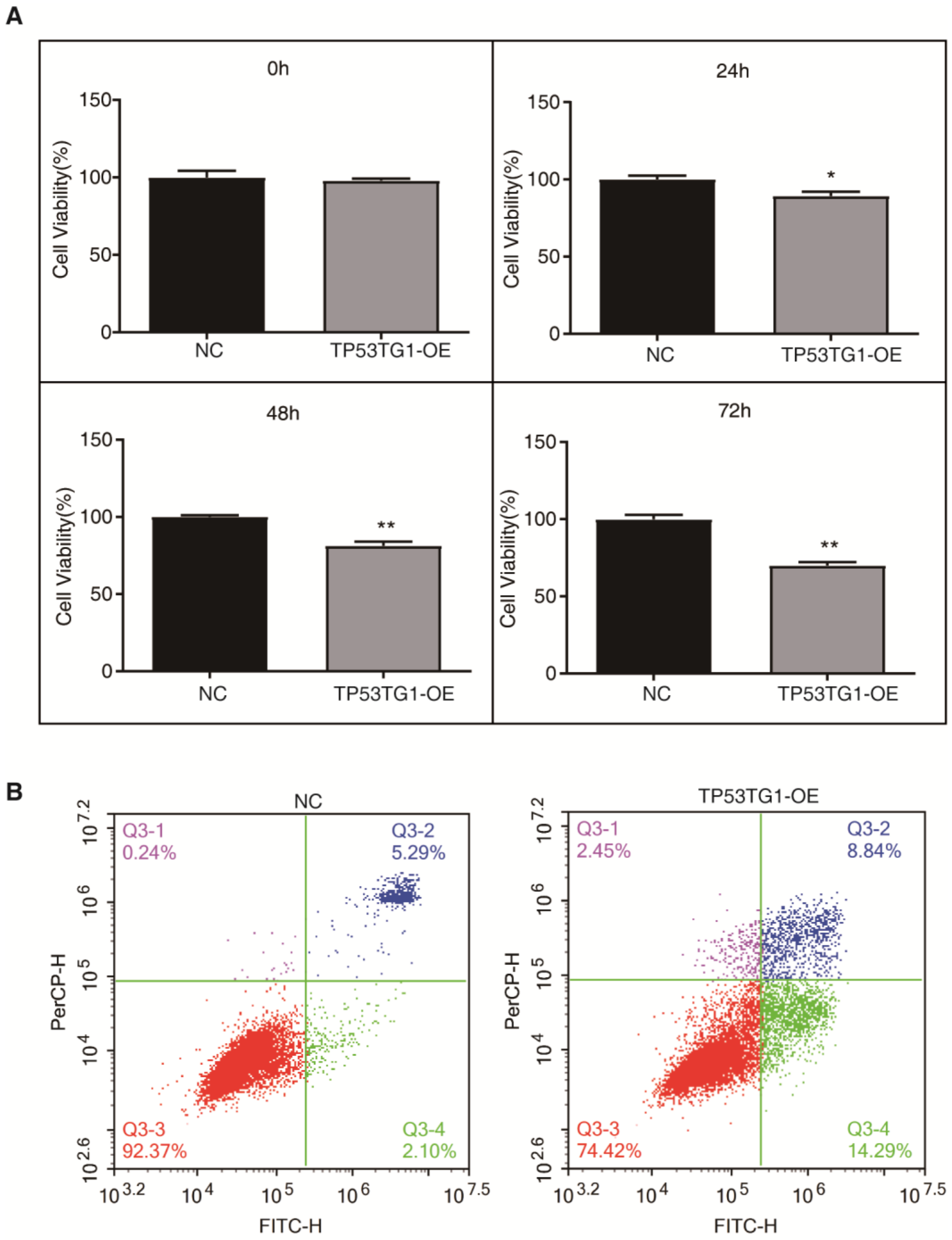

Supplementary Figure 2. TP53TG1 over-expression significantly inhibits proliferation and promotes apoptosis of HeLa cells. **(A)** Cell viability was examined by cell viability assay. (B) Cell viability was examined by cell viability assay.

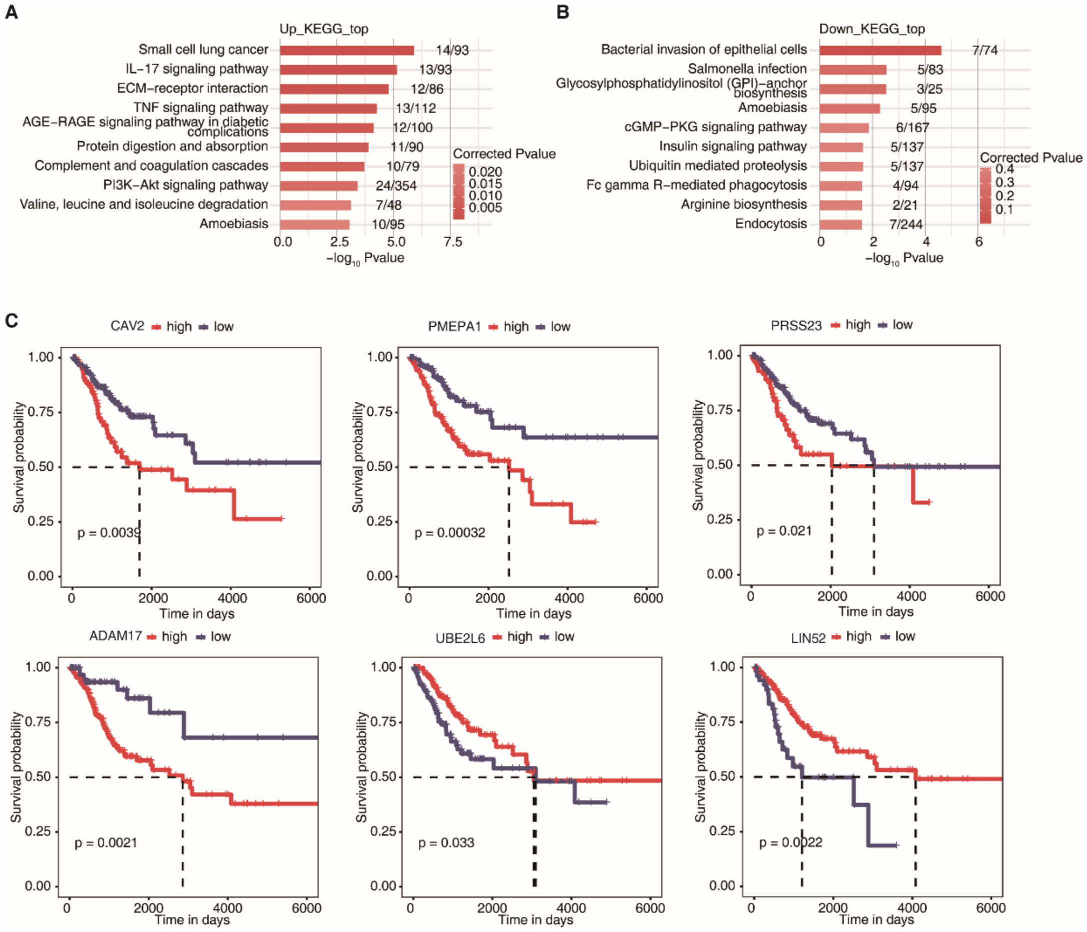

Supplementary Figure 3. TP53TG1 regulates the expression of genes involved in type I interferon signaling pathways and DNA damage responses. **(A-B)** The bar plot exhibiting the most enriched KEGG pathway results of the up-regulated **(A)** and down-regulated **(B)** DEGs. **(C)** Overall survival (OS) analysis of six TP53TG1-regulated DEGs was performed in 293 analyzed CESC patients from TCGA with high versus low expression.

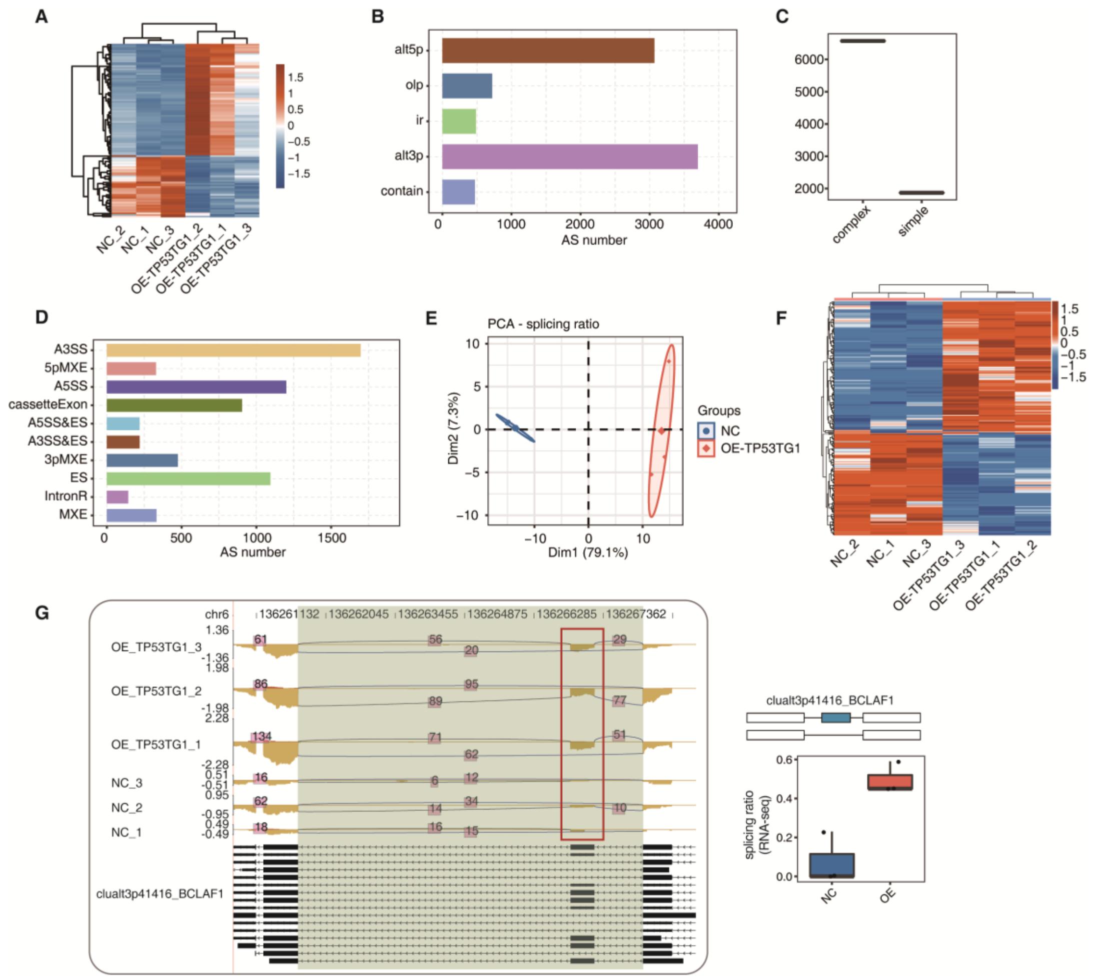

Supplementary Figure 4. TP53TG1 regulates the alternative splicing of genes associated with proliferation and apoptosis process in relation to its regulation of the expression of a large number of RBP genes. (A) The heatmap showing the expression profile of TP53TG1-regulated DE RBPs. (B) Box plot showing all detected AS events by SUVA in the TP53TG1-OE compared with in control group. (C) Box plot showing the number of SUVA AS events that contain SJs involved in two or more different classical splicing events (complex) or in the same classical splicing event (simple). (D) Splice junction constituting AS events detected by SUVA was annotated to classical AS event types. And the number of each classical AS event type was shown in Box plot. (E) PCA base on splicing ratio of TP53TG1-regulated RAS. The ellipse for each group is the confidence ellipse. (F) The heatmap showing the splicing ratio profile of TP53TG1-regulated RAS events with pSAR >= 50%. (G) Visualization of junction reads distribution of clualt3p41416 RAS event located on BCLAF1 in samples from different groups. Splice junctions were labeled with SJ reads number. And altered splice site was marked out with red box. Splicing events model was shown in right-up panel. Splicing ratio profile of RNA-seq and of reverse transcription-qPCR validation in HeLa cells were shown in right-bottom panel.
